# Fatal fulminant necrotizing pneumonia: a case report

**DOI:** 10.1186/1752-1947-8-37

**Published:** 2014-02-05

**Authors:** Dante N Schiavo, Philippe R Bauer, Vivek N Iyer, Jay H Ryu

**Affiliations:** 1 Division of Pulmonary and Critical Care Medicine, Mayo Clinic Gonda, Building 18S, 200 First Street SW, Rochester, MN 55905, USA

**Keywords:** Fungal pneumonia, *Histoplasma capsulatum*, Histoplasmosis, Necrotizing pneumonia

## Abstract

**Introduction:**

Here we present the case of a patient with fatal pulmonary histoplasmosis who presented with extensive necrotizing and cavitating pneumonia. To the best of our knowledge, this case report is the first to describe this presentation in a patient with no known immunosuppression.

**Case presentation:**

A 45-year-old Caucasian woman, a smoker from southeastern Minnesota, presented to our hospital with progressive dyspnea, fatigue and weight loss over the course of several months. Her medical history included type 2 diabetes mellitus, systemic hypertension and chronic opioid use for back pain. She did not have any recent travel history, and she had no unusual hobbies or risk factors for human immunodeficiency virus. When she was admitted to our intensive care unit, she was in hypoxic respiratory failure, thus we intubated her and placed her on mechanical ventilation. A computed tomographic scan of the chest revealed extensive areas of pulmonary necrosis with diffuse bilateral cavitation and lung destruction, which were especially prominent in the upper and middle lung fields. Bronchoalveolar lavage confirmed growth of *Histoplasma capsulatum* as the sole isolated pathogen. No other infectious agents were identified in blood, bronchoalveolar lavage, sputum or urine samples. Her condition worsened over the next 24 to 48 hours, with progressive multi-organ failure in spite of aggressive antibiotic and antifungal therapy. Her family elected to withdraw supportive care, and she died shortly thereafter.

**Conclusion:**

This case demonstrates a novel manifestation of histoplasmosis associated with extensive lung necrosis and cavitation. This report is of particular interest to pulmonologists and intensivists and underscores the importance of maintaining suspicion for mycotic disease in patients who have atypical presentations but live in an endemic area.

## Introduction

Necrotizing pneumonia is a morbid and potentially fatal complication of pulmonary infection characterized by progressive necrosis of lung parenchyma. Most common causative organisms are bacteria, including *Staphylococcus aureus*, *Streptococcus pneumonia* and *Klebsiella pneumoniae. Pseudomonas aeruginosa*, *Haemophilus influenzae*, *Escherichia coli*, *Acinetobacter baumannii*, and anaerobic pathogens have also been reported to cause necrotizing pneumonia [1]. Fungi are a less common cause. For example, *Aspergillus* species can cause necrotizing pneumonia in immunocompromised hosts, particularly those patients who have received hematopoietic or solid organ transplants [2,3]. *Histoplasma capsulatum* can cause asymptomatic disease, acute pulmonary histoplasmosis or chronic pulmonary histoplasmosis. Chronic cavitary pulmonary histoplasmosis can be associated with fibrotic and cavitary changes, but this typically occurs in patients with underlying structural lung disease [4]. Here we present a case of histoplasmosis presenting as rapidly progressive necrotizing pneumonia in a patient with relatively little changes in baseline lung architecture and no known immunosuppression.

## Case presentation

A 45-year-old Caucasian woman, a smoker with a history of type 2 diabetes mellitus, was admitted to our hospital with a 3-month history of progressive exertional dyspnea associated with malaise, cough productive of pink-tinged sputum and a 100-lb unintentional weight loss over the preceding year. She did not use alcohol or illicit drugs, was receiving disability income because of back pain, lived in an apartment with her husband in southeastern Minnesota, did not have pets or animal exposure and had no history of exotic travel.

Her physical examination showed a cachectic, toxic-appearing woman who appeared remarkably older than her actual age. Her vital signs were heart rate 134 beats per minute (bpm), blood pressure 118/74mmHg, respiratory rate 32 breaths/min, oxygen saturation 78% on room air and body temperature 37.0°C. She had a weak cough, and auscultation of the chest revealed diffusely reduced breath sounds bilaterally with faint inspiratory crackles. There was no clubbing. Her cardiac examination revealed tachycardia but no murmurs.

**Figure 1:**
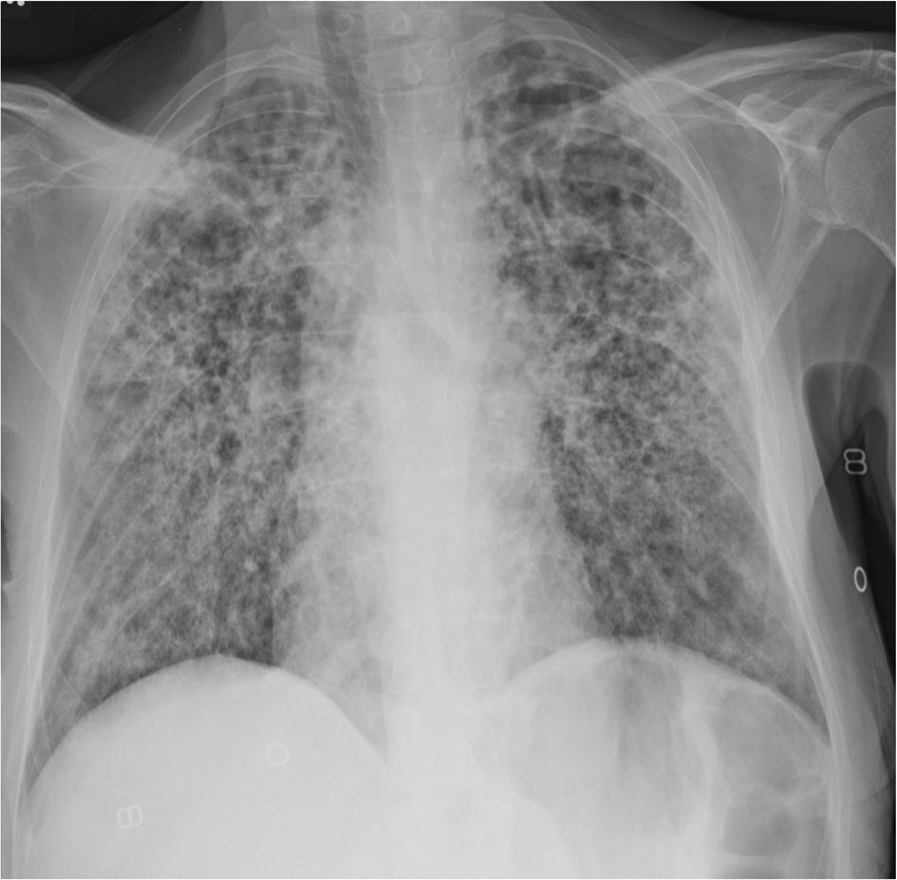
**Anteroposterior chest radiograph taken at time of admission.** This image shows diffuse bilateral interstitial and airspace opacities.

**Figure 2:**
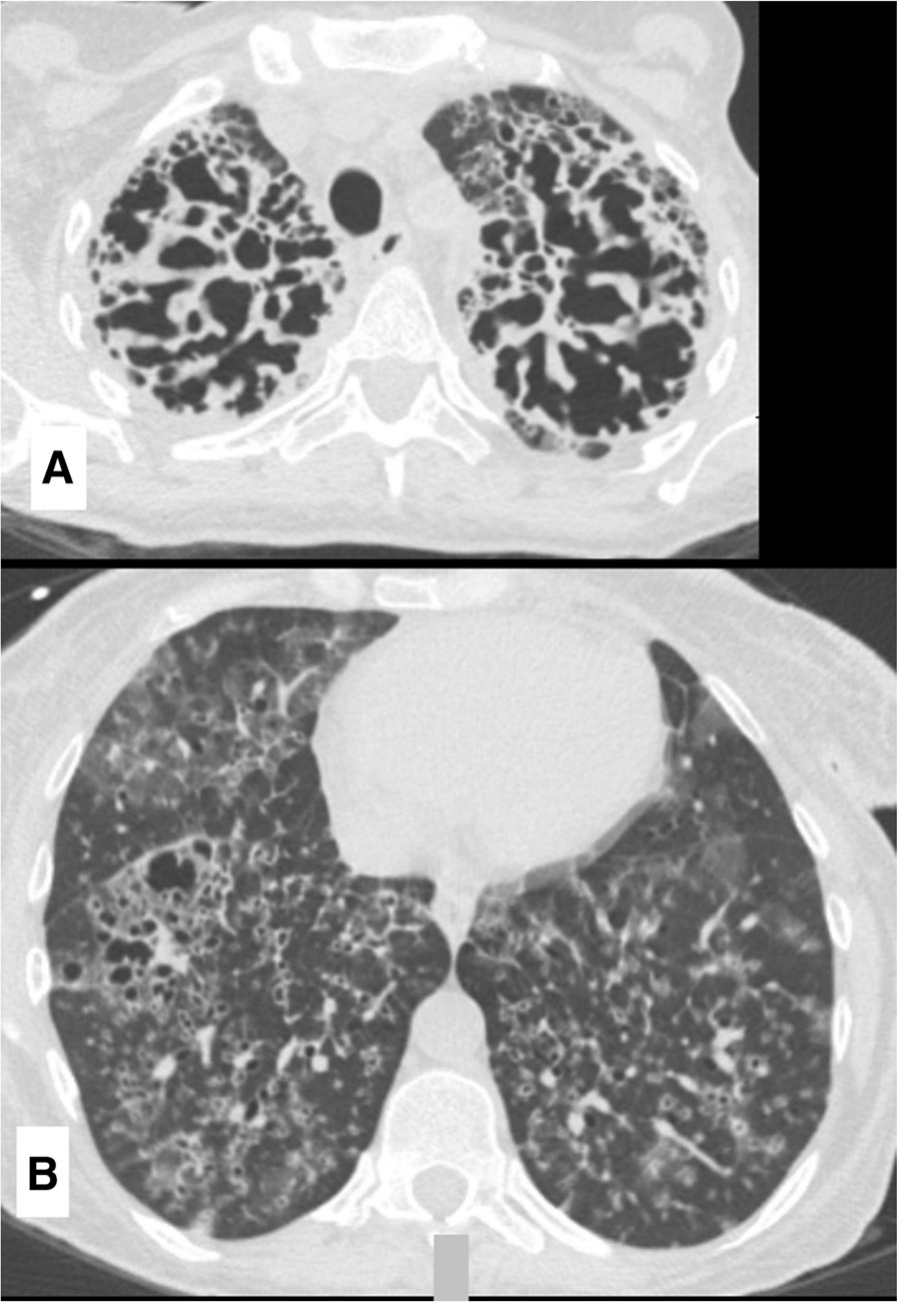
**Computed tomographic scan of the patient’s chest at time of admission. (A)** This image shows bilateral interstitial thickening and airspace destruction at the upper lung zones. **(B)** Ground-glass and cavitary opacities can be observed in the lower lung zones.

**Figure 3:**
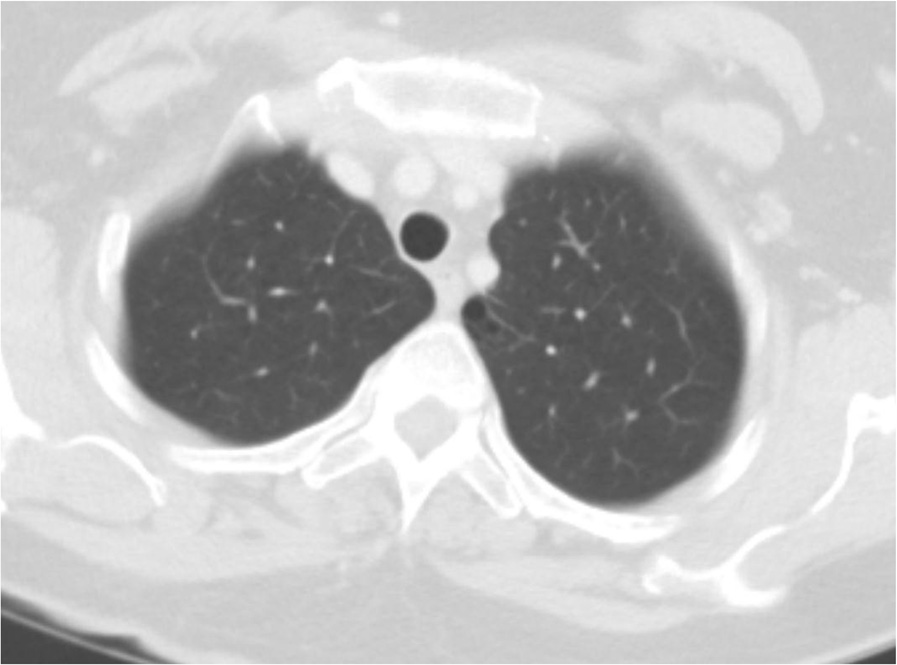
**Computed tomographic scan of the chest taken 9 months prior to admission.** This image shows a small cluster of emphysema at the medial left upper lobe, but otherwise the lung parenchyma is normal.

Laboratory studies showed neutrophilic leukocytosis and low serum potassium, creatinine and albumin levels. The results of an arterial blood gas study on 2L/min of supplemental oxygen were pH 7.38, partial pressure of carbon dioxide in arterial blood 24mmHg, partial pressure of oxygen in arterial blood 71mmHg and bicarbonate 14mmol/L. Urinalysis was unremarkable. Antibody testing for human immunodeficiency virus (HIV) type 1 and HIV type 2 was negative. Serum immunoglobulin (Ig) levels (IgG, IgM and IgA) were within normal limits. The results of serum protein electrophoresis showed no monoclonal proteins, and a connective tissue panel for rheumatologic disease was negative. Chest radiography showed marked, diffuse, bilateral interstitial and alveolar opacities with slight upper-lobe predominance (Figure 1). High-resolution computed tomography (CT) of the chest revealed extensive bilateral interstitial thickening with ground-glass and cavitary opacities associated with parenchymal destruction that was most pronounced in the upper lungs (Figure 2A and 2B). A CT scan of the chest performed 9 months earlier (Figure 3) because of her weight loss was normal, except for subtle centrilobular and subpleural emphysema in the upper lobes.

The patient underwent bronchoalveolar lavage (BAL), and specimens were sent for microbiologic and cytologic analysis. She was empirically started on vancomycin, cefepime, levofloxacin and, later, amphotericin B. Her condition progressively deteriorated after intensive care unit (ICU) admission. She developed septic shock requiring vasopressors (norepinephrine 0.9μg/kg/min and vasopressin 0.03U/min) and progressive respiratory failure requiring mechanical ventilation. Her Sequential Organ Function Assessment Score increased from 1 to 11 by hospital day 3, suggesting a poor prognosis for recovery [5]. The patient’s family decided to withdraw support, and she died on hospital day three.

Post-mortem bacterial cultures of the BAL fluid were negative. Polymerase chain reaction testing for *Pneumocystis jiroveci*, influenza, adenovirus, respiratory syncytial virus, *Legionella* and *Mycobacterium tuberculosis* was negative. The patient’s histoplasma serology showed elevated complement fixation titers to both mycelial phase (1:512) and yeast phase (1:128), and an immunodiffusion assay showed the presence of H and M bands. BAL fungal cultures grew many *H. capsulatum* organisms.

## Discussion

Necrotizing pneumonia denotes infection-related pulmonary consolidation complicated by necrosis or gangrene of the lung tissue [6]. Although the term *necrotizing pneumonia* refers to a non-specific process potentially seen in multiple different lung infections, most cases described in the literature have been attributable to bacterial pathogens. A recent review identified *S. aureus*, *S. pneumoniae* and *K. pneumoniae* as the most common pathogens causing necrotizing pneumonia [1]. Tseng and colleagues [7] conducted a review of 30 pediatric patients with clinical, radiologic and histologic evidence of necrotizing pneumonia. In their series, most patients either had infection with *S. pneumoniae* or *S. aureus* or no causative microorganism that could be identified. In five patients, however, only a non-bacterial pathogen was isolated from lung tissue, including *Mucor* species and *Aspergillus fumigatus*. All patients with non-bacterial pathogens isolated were immunosuppressed. Subsequent studies have also suggested that non-bacterial infectious causes for lung necrosis are more common in immunocompromised patients [8,9]. Our patient was not known to be immunosuppressed. She had no immunosuppressing conditions or medication exposure.

Although *H. capsulatum* is common in the patient’s geographic region, our suspicion for the presence of this organism was initially limited because of the atypical presentation. Chronic pulmonary histoplasmosis can be associated with cavitary changes but is usually seen in older patients and in individuals with significant underlying parenchymal disease [4]. The chest CT in such patients typically demonstrates interstitial infiltrates, along with pre-existing changes of emphysema and foci of cavitation or destruction with thickened walls [10]. Our patient was relatively young. A CT scan of the chest performed 9 months prior to her presentation to our hospital showed little parenchymal change. As such, we felt her presentation would be atypical for fungal pneumonia, and the initial empiric antimicrobial regimen covered primarily bacterial pathogens. After she experienced several hours of clinical deterioration, liposomal amphotericin B was added.

Direct tissue analysis using microscopic stains and cultures for additional organisms could not be performed. However, we think that the many *H. capsulatum* organisms isolated on deep respiratory culture and the very high serology titers strongly implicate this organism as a pathogen that contributed to the patient’s fatal pneumonia.

## Conclusion

This patient’s presentation was unique in that *H. capsulatum* was associated with rapidly progressive lung necrosis in an apparently immunocompetent host. This case highlights the importance of including non-bacterial pathogens in the differential diagnosis of necrotizing pneumonia, particularly in patients who live in areas with common endemic mycoses.

## Consent

Prior to her death, written informed consent was obtained from the patient for publication of this case report and any accompanying images. A copy of the written consent is available for review by the Editor-in-Chief of this journal.

## Abbreviations

BAL: Bronchoalveolar lavage; bpm: Beats per minute; CT: Computed tomography; Ig: Immunoglobulin; lb: pound.

## Competing interests

The authors declare that they have no competing interests.

## Authors’ contributions

DS was involved in the patient’s care, performed a literature review and was the primary author of the manuscript. PB and VI were involved in the patient’s care, performed a literature review and were major contributors to the writing of the manuscript. JR performed a literature review and was a major contributor to the writing of the manuscript. All authors read and approved the final manuscript.
